# Inter-protomer opening cooperativity of envelope trimers positively correlates with HIV-1 entry stoichiometry

**DOI:** 10.1128/mbio.02754-24

**Published:** 2025-02-25

**Authors:** Revansiddha H. Katte, Wang Xu, Yang Han, Xinyu Hong, Maolin Lu

**Affiliations:** 1 Department of Cellular and Molecular Biology, School of Medicine, The University of Texas at Tyler Health Science Center, Tyler, Texas, USA; Rutgers-Robert Wood Johnson Medical School, Piscataway, New Jersey, USA

**Keywords:** HIV-1, virus entry, envelope, trimer, protomer, stoichiometry, cooperativity

## Abstract

HIV-1 entry to host cells is fulfilled by fusion, mediated by surface glycoprotein envelope (Env) trimers upon interaction with host receptors. The entry stoichiometry (T) defining the number of Env trimers required for fusion remains elusive. Stoichiometry was previously estimated using mathematical modeling of infectivity curves of pseudoviruses surface-decorated with heterotrimers containing wild-type and entry-deficient Env. Nevertheless, previous models rarely co-considered inter-protomer opening cooperativity (S, reflecting how CD4-induced conformational changes in one protomer affect the opening of adjacent protomers) and virion trimer number distributions, while experiments were limited to pseudoviruses. Here, we factored these two parameters into our models and included replication-competent virions. We provided simultaneous estimates of T and S under varying trimer number distributions and offered 2D stoichiometry maps for different Env strains. Our results depicted the interplay between viral infectivity and stoichiometry tuned by the number of trimers per virion. The estimates for all tested Env strains were prevalently higher (T ≥ 7 for BG505 or JR-FL, T ≥ 13 for NL4-3) than reported. A high degree of inter-protomer opening cooperativity was observed for the neutralization-sensitive NL4-3, while neutralization-resistant BG505 and JR-FL showed a low to intermediate degree. Entry stoichiometry and opening cooperativity were strikingly positive-correlated, implying tied inter-protomer and inter-Env cooperative interactions. Our findings provided an in-depth view of Env cooperativities during HIV-1 entry.

**IMPORTANCE:**

The sparsely distributed envelope (Env) trimers on the surface of HIV-1 work collaboratively to mediate viral entry into the host, the early step of infection. The number of interacting trimers with host receptors required for entry awaits elucidation. Here, we explored the cooperative interplay among and within Env trimers, shedding light on a previously overlooked dimension of HIV-1 entry. For the first time, we presented distributions of estimated parameters depicting the number of Env trimers and degrees of inter-protomer opening cooperativities using biologically relevant mathematic models combined with virion infectivity measurements. Our results demonstrated that the quantity of required functional trimers positively correlates with inter-protomer opening cooperativity, a feature conserved across various strains. Our findings underscore cooperative behavior as an inherent characteristic of Env dynamics during HIV-1 entry. These insights enhance our understanding of HIV-1 infection mechanisms and could inform strategies for developing effective inhibitors or neutralizing agents.

## INTRODUCTION

Human immunodeficiency virus type 1 (HIV-1) causes chronic infections in humans and can lead to AIDS if left untreated. The first step of HIV-1 entry into a host CD4^+^ target cell is viral membrane fusion between the viral and target cell membranes (1, 2). Fusion is mediated by HIV-1 surface envelope (Env) glycoprotein upon interactions with cell-surface receptor CD4 and coreceptor CCR5 or CXCR4 molecules (3–5). Env is a trimer, and each protomer comprises surface receptors/coreceptors-binding subunit gp120 and the transmembrane subunit gp41 (3, 6–9). In response to CD4 binding, Env undergoes a multi-step gp120 opening to expose structural components essential for coreceptor binding (2, 10–14). Interactions with coreceptors further trigger serials of structural rearrangements in gp41 to eventually form a stable six-helix bundle, which is hypothesized to drive viral and cellular membranes together for fusion (2, 15). The cooperative opening of three protomers in an Env upon interactions with one, two, and up to three CD4 molecules has recently come into low- and high-resolution pictures as structural technologies advance (16, 17). However, the downstream fusion events are still largely unknown. It remains elusive how coreceptors interact with an open Env trimer, what conformational changes gp41 undergo after coreceptor binding, and how many Env trimers work in concert in a productive fusion event leading to cell entry. The entry stoichiometry (T), the number of interacting Env trimers with host receptors required for viral membrane fusion or making an HIV-1 virion infectious (by definition), is awaiting clarification. The T values directly affect the contribution of virions in populations to infection and sensitivity to inhibition or antibody neutralization (18, 19).

A widely adopted approach to estimate HIV-1 entry stoichiometry relies on mathematical modeling in combination with the infectivity data of pseudotyped virions surface-decorated with heterotrimers (20). The pioneering work used an Env mutant with an entry-deficient phenotype (21), and this strategy has been used in later studies (19, 22–25). Co-transfection of virus-producing cells with different fractions of plasmids encoding functional and function-impaired entry-deficient Env allowed the generation of virus stocks containing different compositions of heterotrimers. The entry stoichiometry was retrieved by applying mathematical models to fit infectivity curves of virions carrying those trimers. The initial application of this methodology opened this research direction (21), even though the result of a single Env trimer (T = 1) adequate for HIV-1 entry was later questioned by other studies using various models (19, 22–25). Stochiometric estimates of eight trimers (23), four to five trimers (24, 25), one to two trimers (22), and one to seven dependent on specific strains (19) were suggested by different research groups. The mathematical models used in these studies weigh differently on how the proportion of heterotrimers translates into relative virus infectivity based upon dissimilar assumptions, in part explaining the discrepancy in estimated T numbers. Another important factor contributing to the lack of consensus on T estimates is the unknown distributions of functional Env trimers on virions. In general, approaches used in these studies (19, 21–25) differ in either model parameters or heterotrimer virion infectivity or both, primarily including (i) one or a few constant numbers of trimers or distributions of trimer numbers per virion; (ii) fusion-defective Env with modifications at the gp120/gp41 cleavage site (R508S/R511S), gp41 fusion peptide (V513E), gp120 V3 region, or gp120 CD4-binding interface; (iii) infectivity of a single Env strain or more inclusive multiple strains; (iv) basic model or more sophisticated mathematical models. Some models used a constant number of trimers per virion, while others did not account for inter-protomer opening cooperativity, where CD4-induced structural changes in one protomer affect the conformational opening of adjacent protomers during fusion activation. In prior studies, replication-incompetent pseudovirions carrying full-length Env (not truncated version) were used for infectivity measurements, but replication-competent virions (capable of multi-round infection) were not used.

In this work, we revisited the topic using a CD4-binding inactive D368R mutant Env, an entry-deficient Env closely related to the definition of entry stoichiometry (Env–CD4 interactions). We included replication-competent and pseudotyped virions for generating infectivity curves. We estimated stoichiometry distributions using new models co-considering inter-protomer opening cooperativity and functional trimer distributions on virions. Notably, in this study, inter-protomer opening cooperativity (S), or opening cooperativity for short, is used interchangeably to describe the cooperative opening of the three protomers in an Env trimer, in which the opening of one protomer will affect that of neighboring ones to different extents. In our study, the estimated map of stoichiometry distributions varied across different tested Env strains dictated by trimer number distributions. T estimates were prevalently higher than previously reported and exhibited a positive correlation pattern with inter-protomer opening cooperativity. The neutralization-sensitive NL4-3 Env showed a strong opening cooperativity (S = 3, a single wild-type protomer is sufficient to compensate for the entire trimer opening) and a high stoichiometry (T ≥ 13). By contrast, neutralization-resistant strains BG505 and JR-FL exhibited hard-to-open features (S = 1 or 2) and stoichiometry of no less than seven trimers. A more open L193A JR-FL Env did not appear to divert the opening cooperativity notably but altered the stoichiometry. Our results highlight the highly tied inter-protomer opening and inter-Env cooperativity and offer a map of possible entry stoichiometry for different strains under a realistic broad range of virion trimer number distributions.

## RESULTS

### The interplay between infectivity, virion trimer numbers, and entry stoichiometry

**Fig 1:**
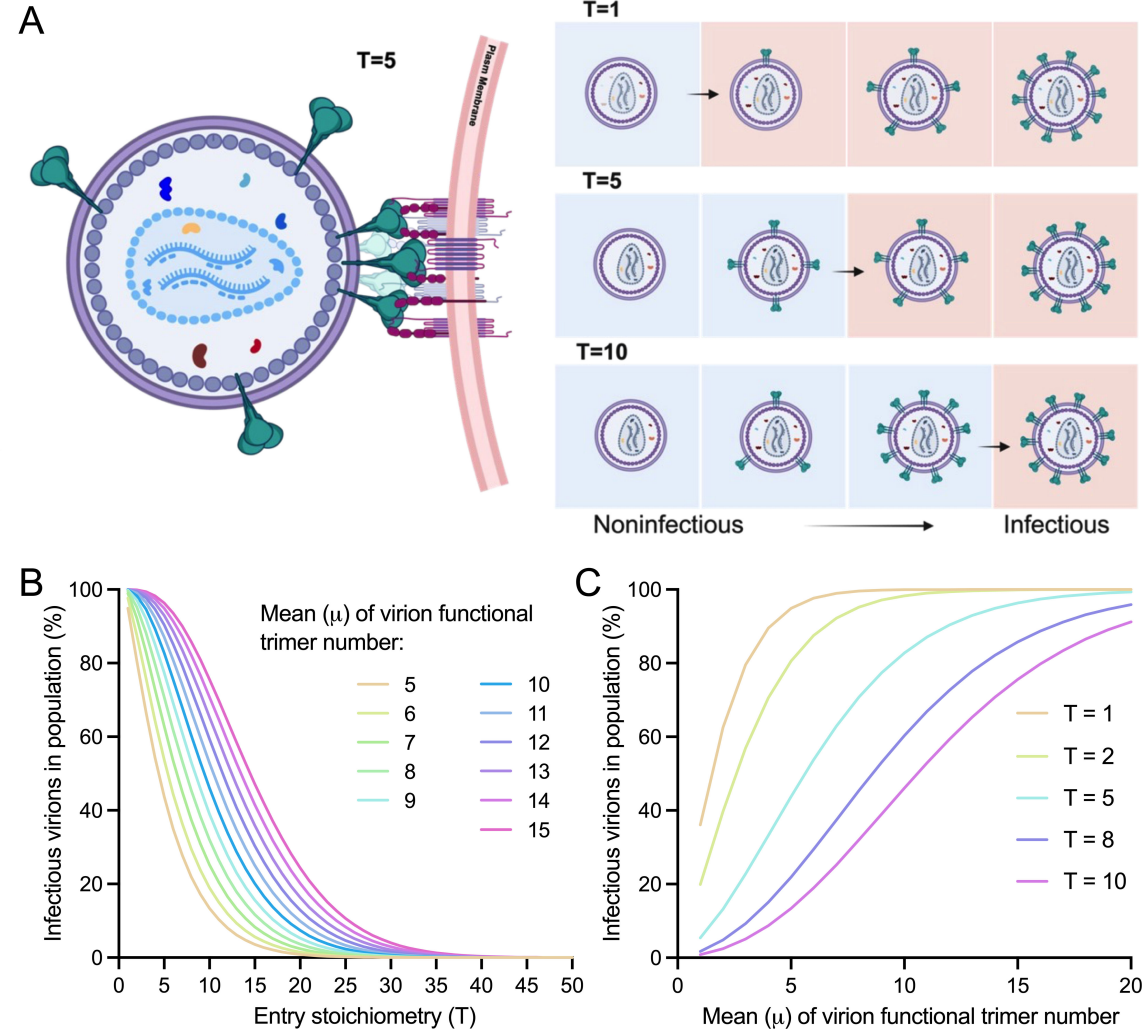
The interplay between HIV-1 virus population infectivity, entry stoichiometry, and virion functional Env trimer number distributions (defined by mean). (**A**) Schematics depicting a few Env trimers (as an example) on a virion during fusion (left) and how entry stoichiometry determines infectious virions or noninfectious virions in the population (right). (**B**) Theoretical predictions of viral population infectivity (%) as a function of entry stoichiometry (T) under different virion average numbers of functional Env trimers (μ, mean). Functional Env trimer distributions are assumed to follow discretized Beta with mean μ and μ-defined variance δ^2^ (for simplicity). See Methods for details. (**C**) The correlation between infectious virions in population and virion functional trimer number distributions under different entry stoichiometry (T).

To demonstrate the biological significance of this study, we first predicted how virion trimer distributions and entry stoichiometry are linked to virus population infectivity. An intriguing study captured the remarkable negative correlation between viral infectivity of 11 different Env strains and their estimated T values (19). This observation implies that Env strains that require fewer trimers for entry are more infectious or have higher fusion potentials than those that require more trimers. The negative correlation is intuitive and is likely accurate if there is no difference in virion trimer distributions among tested Env strains. Of note, this correlation can be shaped by varying trimer number distributions to different extents, as indicated in our modeling results (Fig. 1). Entry stoichiometry T is presumably the cutoff value between infectious and non-infections virions in the population (viral stocks), that is, only virions having no less than T trimers can contribute to virus infectivity (Fig. 1A). For a population of virions carrying a defined distribution of Env trimers, the cumulative possibilities of qualified virions (having ≥T trimers) at any given T can be further translated into the percentages of infectious virions in the population. We, therefore, performed mathematical analyses to dissect the interplay between virus infectivity, T, and virion trimer distributions (Fig. 1B and C). The average number (mean) of trimers on virions was assumed to confine variations in the model, and functional or unfunctional trimers were not differentiated for simplicity (all trimers are functional in this case). Under any given trimer distribution defined by a mean, high T values rendered virions with fewer than T trimers not infectious, and our prediction showed the resulting low percentage of infectious virions in the population and vice versa (Fig. 1B). The extent to how changes in T values affect the percentage of infectious virions varied and seemingly diminished with increasing mean trimers per virion (Fig. 1B). Given Env strains with the same T, those strains with averagely more trimers on virions will have a higher percentage of infectious virions in population than those with less (Fig. 1B), thus are more advantageous in terms of infection. It can be further transformed to predict the extent of T dependence of virus infectivity, varying with the mean number of trimers per virion (Fig. 1C). If Env strains have the same mean number of trimers per virion, those of a higher T (high cutoff value) will exhibit a lower percentage of infectious virions in population, as shown in Fig. 1C. By contrast, those of a lower T are more superior in infecting cells. It is easier to imagine that strains only requiring one trimer for entry (T = 1) are highly infectious clones, in which infectious virions constitute a considerable percentage of the virion population even if each virion, on average, only has one Env trimer (Fig. 1C). Our predictions of the tri-interplay here are consistent with experimental observations of reverse correlation between entry stoichiometry and viral infectivity (19) under the condition of similar virion trimer number distributions across different tested strains.

### CD4-binding defective Env constituted in heterotrimers on replication-competent or pseudotyped virions

**Fig 2:**
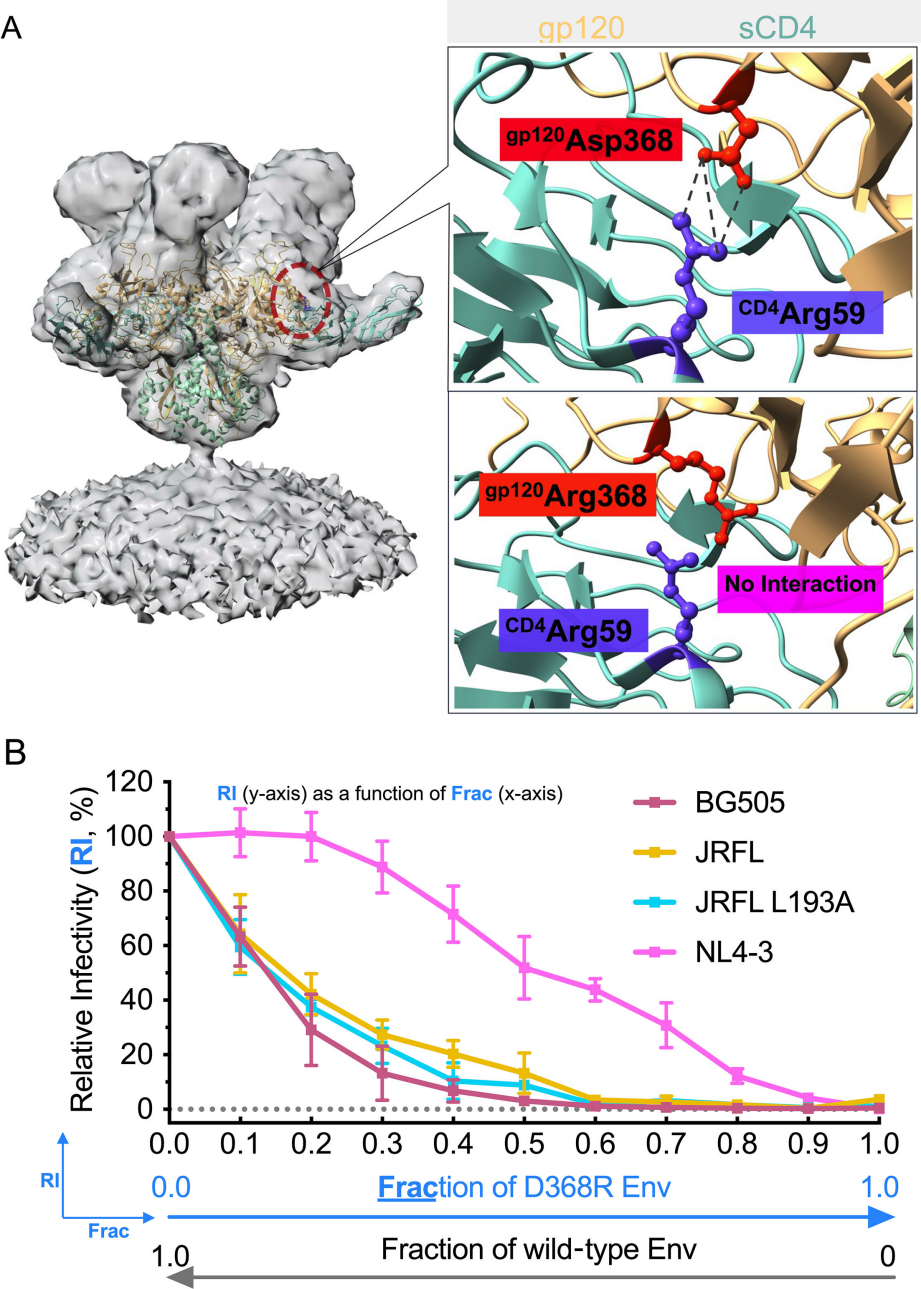
The use of CD4-binding-deficient D368R Env in estimating HIV-1 entry stoichiometry. (**A**) Illustration of binding critical electrostatic interaction between residues 368D in Env gp120 and 59R in CD4 (top) and its elimination by a D368R modification (bottom). The interaction is highlighted on the Env–CD4 complex structure (PDB: 5VN3) fitted into the electron density map (EMD: 21411). D368R modification eliminates the CD4 binding to Env, rending Env fusion incompetent. (**B**) Relative infectivity of replication-competent and pseudotyped HIV-1 virions as a function of the fraction of D368R mutant Env. The infectivity of BG505 or NL4-3 virus stocks expressing the indicated fraction of D368R was evaluated using replication-competent virions, whereas that of JR-FL was using pseudotyped. Infectivity was measured on TZM-bl cells and normalized to that of virus stocks containing solely wild-type Env. The data represent the mean ± SD of two to four independent replicates.

The choice of entry-deficient Env constituted in heterotrimers on virions and the use of replication-competent or pseudotyped virions contributed to experimental inputs in estimating entry stoichiometry, which generally involves infectivity curves of heterotrimer virions in combination with mathematical modeling (20). In heterotrimer virions, entry-deficient mutant and wildtype Env trimers are present in different compositions. We first chose the CD4-binding defective D368R mutant as the entry-deficient Env. A salt bridge formed between 368D_Env_ of Env and 59R_CD4_ of CD4 is a key point of contact during Env–CD4 interactions (Fig. 2A). The alternation of the aspartic acid (D) to arginine (R) at position 368, named D368R, disrupts the formation of this critical salt bridge and makes Env defective in CD4-binding (Fig. 2A) (5, 26). In other words, the CD4 binding of D368R Env is blocked, which is consistent with our observations of baseline infectivity signal ~0% for virions carrying 100% D368R Env normalized to virions carrying 100% wild-type Env (Fig. 2B). Entry stoichiometry is the number of trimers required to interact with host cell receptors to mediate virus entry. Thus, the Env–CD4 contact disrupting feature of D368R makes it closely relevant to entry stoichiometry estimates from the CD4-induced fusion activation viewpoint. Another merit of using D368R Env is its comparable levels of expression and virion incorporation to the wild-type Env, akin to our previous evaluation of three Env strains (10). The same three Env strains were evaluated in this study.

Replication-competent virions have not been used in previous entry stoichiometry studies. Here, we used heterotrimer replication-competent and pseudotyped virions as virus stocks for infectivity measurements (Fig. S1). We included Env of three strains, neutralization-sensitive laboratory-adapted NL4-3, and two neutralization-resistant primary strains: BG505 and JR-FL. A more open JR-FL L193A was also included for comparison. Replication-competent virions decorated with Env_BG505_ heterotrimers of wild-type and D368R protomers were prepared by transfecting HEK293T cells with a mixture of HIV-1_Q23_ Env_BG505_ wild-type and D368R at serial ratios (fractions of D368R) along with a luciferase reporter gene. Replication-competent NL4-3 virions and pseudotyped JR-FL virions were generated similarly. Infectivity curves of those heterotrimer virions were determined on TZM-bl cells based on luciferase activities. Measured infectivity was further displayed as a function of the fraction of D368R (Fig. 2B), which reflects the stoichiometry of Env–CD4 interactions necessary for cell entry that requires appropriate modeling.

### Factoring inter-protomer opening cooperativity and virion trimer distributions in stoichiometric parameters

**Fig 3:**
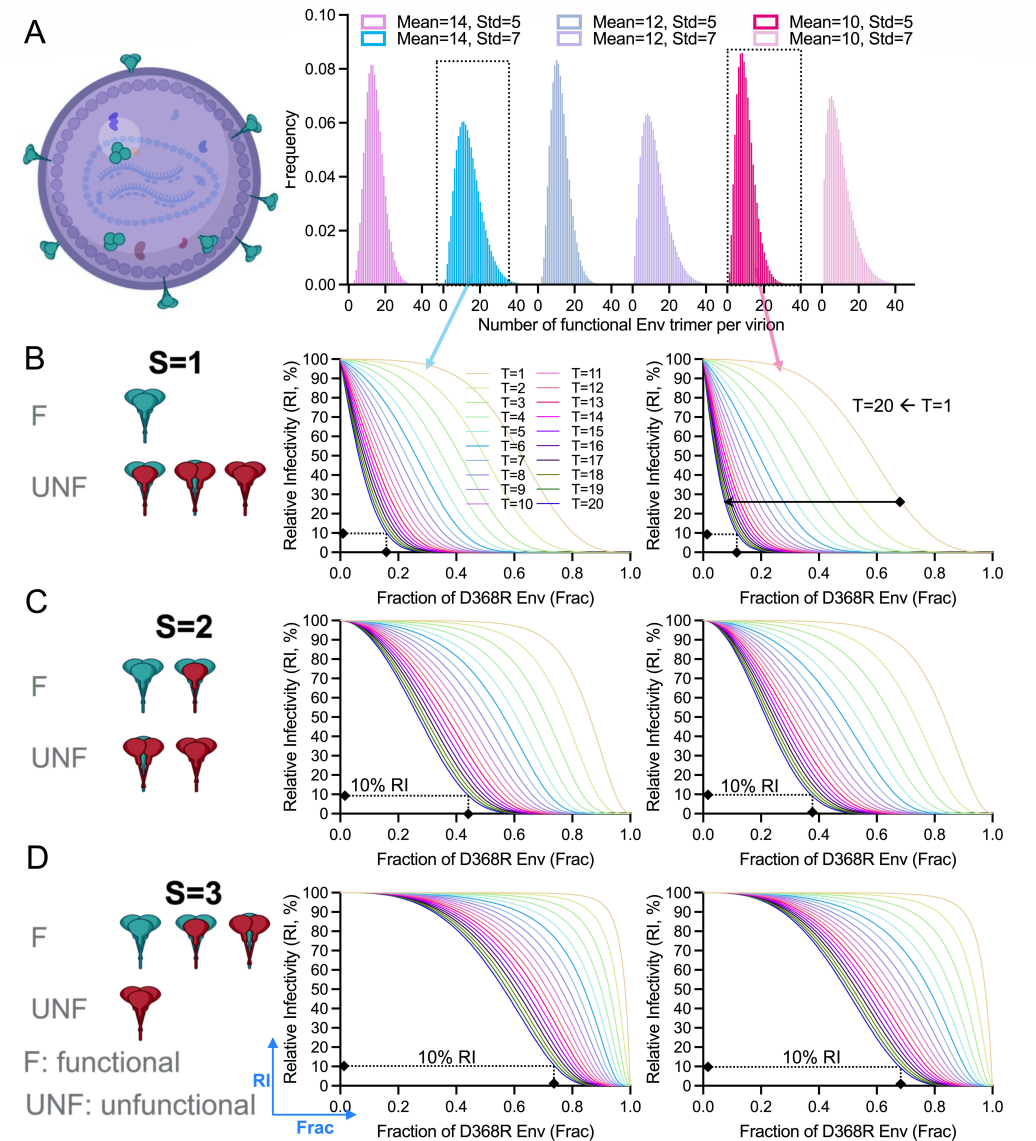
Functional Env trimer numbers on virions and Inter-protomer opening cooperativity (S) factored in modeling the entry stoichiometry (T) estimations. (**A**) Different distributions of functional Env trimers on virions modeled by discretized Beta (mean, std) distributions. Mean is the average number, and std is the standard deviation of functional trimer numbers across virions. (B–D) Model predictions of relative viral infectivity (RI) as a function of the fraction of CD4-binding inactivated D368R mutant Env (Frac) under different S. S = (1, 2, or 3) is the case that (1, 2, or 3) D368R protomer in a trimer renders the trimer unfunctional for fusion. S of 1, 2, and 3 reflects low, intermediate, and high inter-protomer opening cooperativity or compensation, respectively. (**B**) Model predictions of relative viral infectivity (RI) in the case of S = 1. Exampled by two trimer distributions, predicted curves vary with the number of T ranging from 1 to 20 (as indicated by different line colors). (**C and D**) Model predictions as in (**B**) in the case of S = 2 (**C**) and S = 3 (**D**). Predicted curves of RI as Frac (B–D) changes with different S, T, and functional trimer distribution Beta (mean, std). In B–D, the corresponding fraction of D368R under RI of 10% was used to highlight the curve differences.

We then generated mathematical models to factor in the two often non-coincidentally considered stoichiometry parameters, opening cooperativity (S) and virion trimer number distributions. Similar to the approach used in previous studies (19, 23), we adapted discretized Beta distributions to model the distributions of functional Env trimers on virions, and the shape of Beta distribution was confined by a pair of mean and deviation/variation (Fig. 3A). Different from their approaches, our model is not limited to any given mean number of trimers. Instead, our model screens for any combination of mean and deviation within a wide realistic range and yields a matrix of T estimates, not just a single or a few numbers (Methods and Materials). Another difference is accounting for inter-protomer functional compensation, reflecting inter-protomer opening cooperativity in our case. We differentiated the degree of functional compensation (S) with reduced complexity to three levels: S = 1, S = 2, and S = 3. S represents the number of CD4-binding-defective protomers within a trimer necessary to render the Env unfunctional (Fig. 3B through D). S of 3 refers to the scenario that one functional protomer is sufficient to compensate for the functional loss of the other defective protomers in a trimer. The defective D368R protomer is CD4-binding eliminated and thus is CD4-induced opening incompetent if there is no cooperativity from neighboring protomers. Therefore, S of 3 means that one single CD4-bound protomer can open the entire trimer, reflecting the high level of opening cooperativity and suggesting an easy-to-open feature of Env trimers. By contrast, S of 1 and 2 suggests low and intermediate opening cooperativity, respectively, with a decreasing hard-to-open trait. We acknowledge the inherent complexity of the hypothesis that a single CD4-bound protomer can open the entire trimer due to high inter-protomer cooperativity, as well as the simplicity of our model (S = 1, 2, 3) without considering the gradual effects of S. Despite these limitations, this hypothesis is consistent with structural studies and aligns with the prevailing understanding in the field. We further used our simplified model to simulate infectivity curves of heterotrimer virions with T values ranging from 1 to 20, based on two exampled distributions of virion trimer numbers and different degrees of S (Fig. 3B through D). For any individual infectivity curve, if the distribution of functional trimer numbers on virion is known, T estimates vary significantly with the degree of S. Similarly, if S is given, T estimates change with the distributions of virion trimer numbers. Our results demonstrated the dependence of entry stoichiometry on virion trimer number distributions and opening cooperativity.

### NL4-3 demonstrates high opening cooperativity but requires more trimers for entry

**Fig 4:**
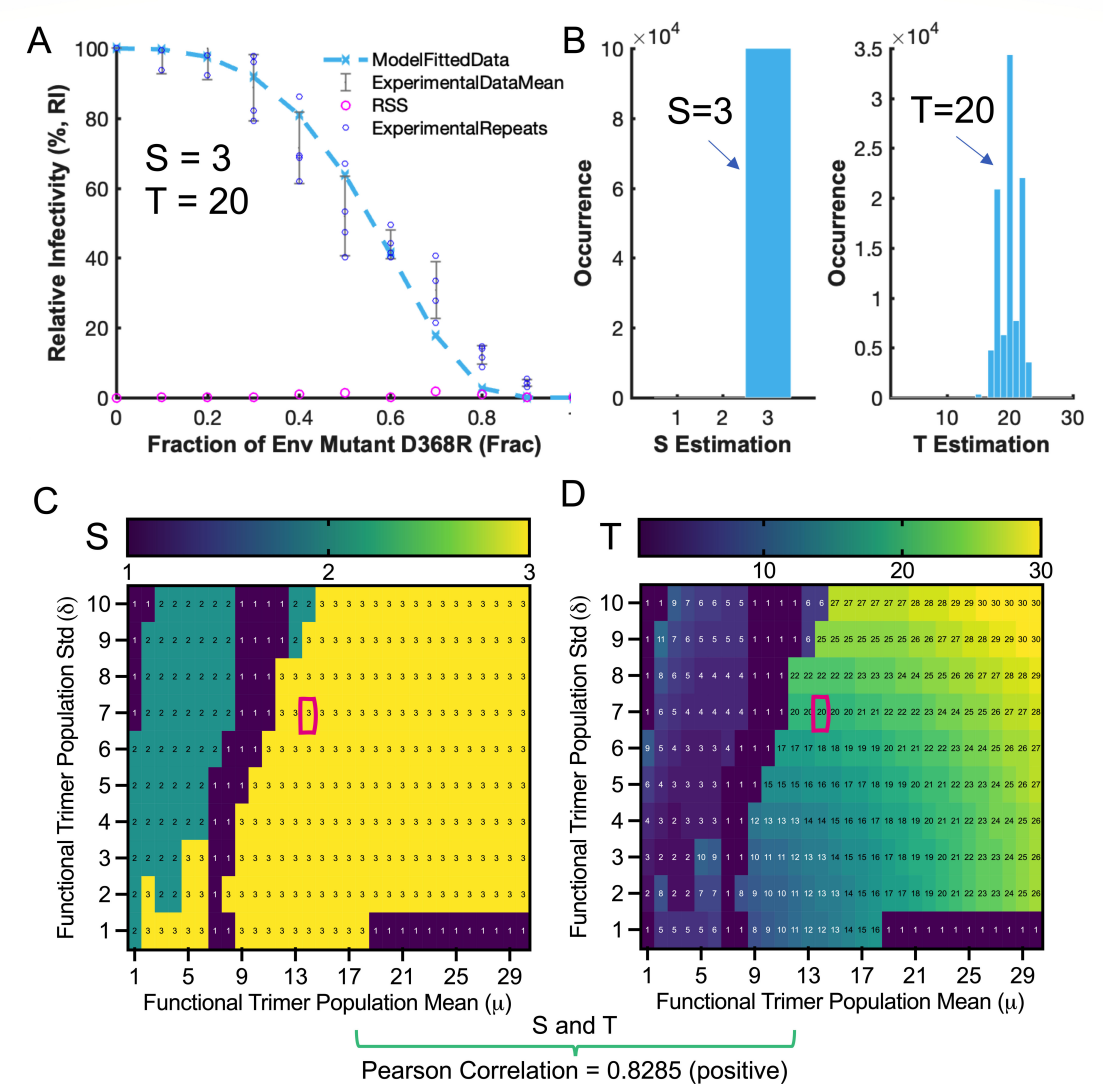
High inter-protomer opening cooperativity (S = 3) and a relatively large T number (≥13) of neutralization-sensitive laboratory-adapted strain Env_NL4-3_ trimers. (**A**) Estimates of S = 3 and T = 20 for Env_NL4-3_ from model fits of relative viral infection of replication-competent HIV-1_NL4-3_ over the fraction of D368R Env_NL4-3_ mutant, defined by experimentally evaluated trimer distribution of Env_NL4-3_ (19). RSS (residual sum of squares) is a statistical evaluation of the goodness of model fitting of data. The smaller the RSS is, the better the model fit is. (**B**) Bootstrap validation of S and T estimations (100,000 replicates) in (**A**). (**C and D**) Heatmap displays of S (**C**) and T (**D**) estimates (colormap) with varying Env_NL4-3_ trimer distributions (mean-μ, *x*-axis; std-s, *y*-axis) on virions. High opening cooperativity (S = 3) dominates on the S heatmap. T estimates range prevalently no less than 13 if the average number of trimers is the reported 14. Values in the red boxes correspond to S/T estimates presented in (**A**). The linear correlation between estimated S and T exhibits a strong positive correlation, with a Pearson correlation coefficient of 0.8285.

With our mathematical models in place, we then started to estimate S and T for the NL4-3 strain by model-fitting the heterotrimer replication-competent NL4-3 virion infectivity curve (Fig. 4). The laboratory-adapted NL4-3 was experimentally determined to have a rounded average of 14 trimers on virion with a standard deviation of 7 (19). This information resulted in an estimate of S = 3 and T = 20 (Fig. 4A), which was further validated by running a bootstrap of 100,000 repeats (Fig. 4B). The number of 20 trimers for NL4-3 required for entry is much larger than the previously reported four to seven trimers (19), which did not consider S. The differences in modeling and experimental settings likely explain the inconsistency in estimated T values. We then relaxed the pre-defined trimer distributions and set the mean number of trimers to range from 1 to 30 with a deviation from 1 to 10 (variance from 1 to 100). HIV-1 is known to have a sparse number of Env trimers on the surface, with likely 5–15 trimers per virion (27–29). While the exact distributions of virion trimer numbers are lacking, our setting of limits/boundaries is broad enough to include most virions, if not all. We then explored the estimates of S and T within the range and generated matrices of S and T. S matrix showed the dominance of S = 3 in areas where the mean of nine or more trimers per virion (Fig. 4C). The S heatmap revealed the possibility of S = 1 or 2 in areas where virions have a smaller mean number of trimers (Fig. 4C). The T matrix displayed as a heatmap revealed a spread distribution of T values (Fig. 4D) that increases with increasing number of trimers per virion. If the number of 14 trimers per virion was the case, the number of NL4-3 trimers required for entry is no less than 13 in most cases. Instead of giving a definite number, T distributions obtained from considering various virion trimer numbers provided a map of routes toward reasonable approximation. We also speculated a possible correlation between S and T by visually comparing their heatmap presentations (Fig. 4C and D). The correlation analysis confirmed our assumption and revealed a highly positive linear correlation of 0.8285 between S and T (Fig. 4C and D).

### The positive S–T correlation persists for BG505 Env, which displays a difficult-to-open trait

**Fig 5:**
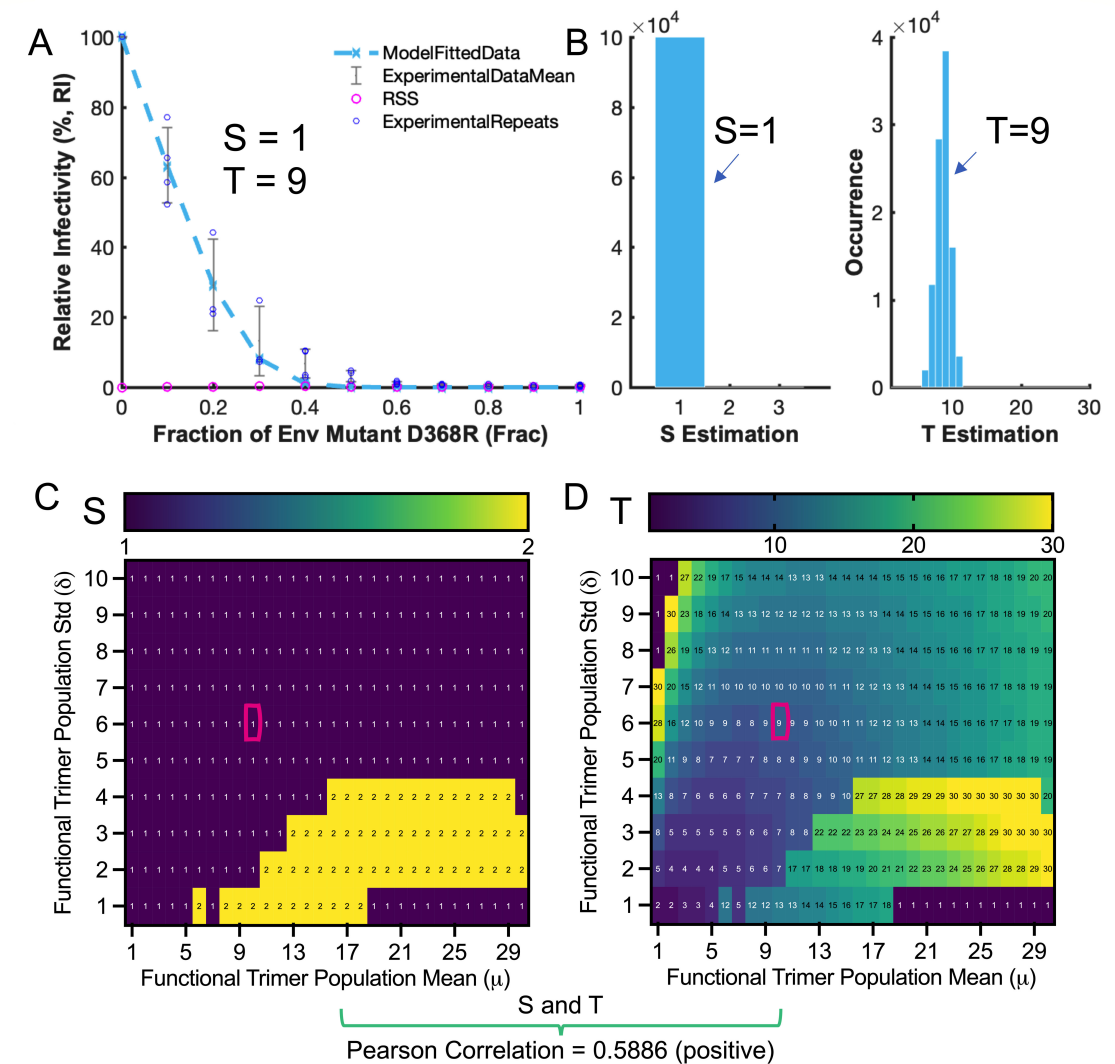
Low and intermediate inter-protomer opening cooperativity (S = 1 or 2) of neutralization-resistant primary strain Env_BG505_ trimers. (**A**) Estimates of S = 1 and T = 9 for Env_BG505_ from the infectivity curve of replication-competent HIV-1_Q23 BG505_ over the fraction of D368R Env mutant, given experimentally defined trimer distribution of Env_BG505_ (19). (**B**) Bootstrap validation of Env_BG505_ S and T estimations (100,000 replicates) in A. (**C and D**) Heatmaps of S (**C**) and T (**D**) estimates, with varying Env_BG505_ trimer distributions on virions. Low and intermediate opening cooperativity (S = 1 and 2) prevails on the S heatmap. T estimates range prevalently larger than seven using the reported average mean of 10. Values in the red boxes correspond to S/T estimates in A. The linear correlation between estimated S and T exhibits a strong positive correlation, with a Pearson coefficient of 0.5886.

We then explored whether the positive correlation persisted for naturalization-resistant primary Env strain BG505, a popular clinically relevant strain widely used in recent vaccine designs (30, 31). The trimer number distribution of BG505 was previously determined by enzyme-linked immunoisorbent assay (ELISA) experiments with a rounded mean of 10 trimers and a confined deviation by the mean (19). Given this input, the fitting of infectivity curves of replication-competent D368R heterotrimer HIV-1_Q23_ Env_BG505_ yielded an estimate of S = 1 and T = 9 with bootstrap validation (Fig. 5A and B). The S of 1 indicates low opening cooperativity, the dominance of which was observed when trimer number distributions were unconstrained (Fig. 5C). Of note, the S of 2 is not exclusive from our observations. Instead, S of 2 prevails in areas of virions carrying, on average, more than eight trimers under low trimer number variations across virions (deviation ranging from 1 to 4). S of 3 was not observed. Altogether, our results strongly implied that BG505 Env trimer is hard to open with low or intermediate inter-protomer opening compensation. The estimated T of 9 is larger than the reported T of 2 or 3 for BG505 (19). As explained earlier, the differences in models (factor S or not), virions (replication-competent or pseudotyped), and mutant (CD4-binding blocking directly relevant or not) could contribute to a diverted estimate of T. Nevertheless, within a broad range of trimer number distributions (mean from 1 to 30 and deviation from 1 to 100), estimates of T for BG505 were rarely below the number of 3 (Fig. 5D), suggesting a likely underestimation of the required BG505 Env trimer for entry in previous studies. Even if the mean of 10 BG505 trimers per virion is more or less accurate, all stoichiometric estimates are no less than 7 (T ≥ 7) on the heatmap (Fig. 5D). The smaller numbers of S and T for BG505 compared with those of NL4-3 are evident (Fig. 4 and 5). We then performed a linear S–T correlation analysis for BG505 to check if the positive correlation observed in NL4-3 persisted. In agreement with the results of NL4-3, BG505 Env also exhibited a positive S–T correlation with a coefficient of 0.5886. The positive S–T correlation appears to be an intrinsic feature of Env that is not tied to specific strains.

### More open JR-FL L193A Env requires more trimers for entry than predominantly closed wildtype

**Fig 6:**
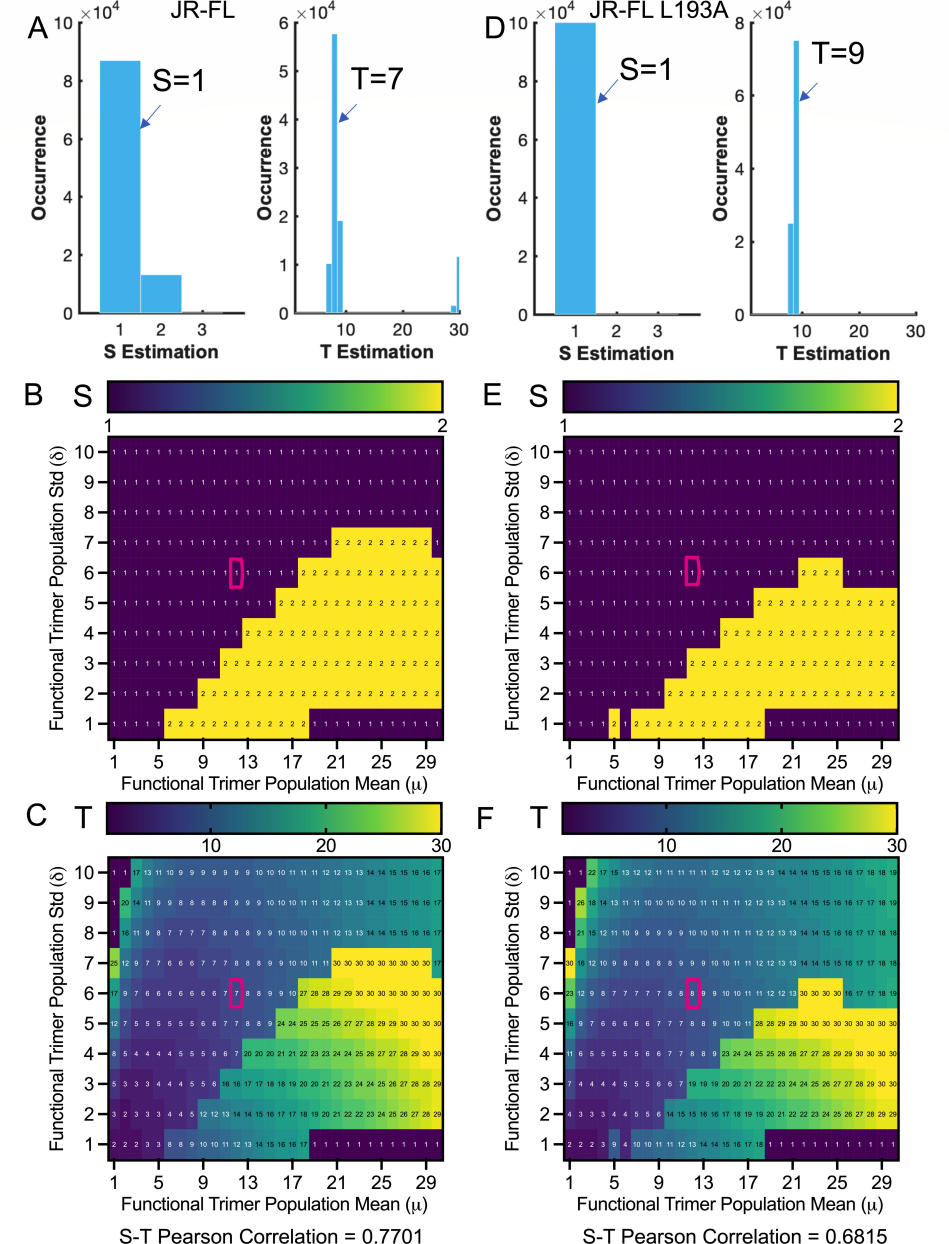
The inter-protomer opening cooperativity is largely retained between Env_JR-FL_ and the more open Env_JR-FL_ L193A, while entry stoichiometry differs. (**A**) Bootstrap valuation of (S = 1, T = 7) for neutralization-resistant primary strain Env_JR-FL_. Infectivity data of pseudotyped virions and model fittings are provided in Fig. S2. (**B and C**) Heatmaps of S (**B**) and T (**C**) estimates with varying Env_JR-FL_ trimer distributions on virions. The T estimates in the heatmaps may have errors of (±1) due to the non-integer standard deviation in the reported distribution. (D–F) Experiments as in (A –C) for more open Env_JR-FL L193A_ trimers. At the population level, T estimated for more open Env_JR-FL_ L193A is larger than the wild type.

We also estimated and compared the S and T of two JR-FL Env, a closed wild type and a more open L193A, using modeling fitting of infectivity curves of heterotrimer pseudotyped virions (Fig. 6; Fig. S2). The reasons for using JR-FL here involve the inclusiveness of pseudotyped virions and the involvement of more Env strains. L193A is an Env variant in which one of the key residues in gp120 V1/V2 that restrains Env opening is altered, and thus L193A Env has a propensity to sample more downstream open conformations compared to predominantly closed wild type (32, 33). The reported mean number of 12 of JR-FL with mean-defined variance (19) yielded an estimate of S = 1 and T = 7 for wild-type Env (Fig. 6A; Fig. S2). Within a broad range of timer number distributions, estimates of S distribution (Fig. 6B) largely lay with 1 or 2, showing a similar opening cooperativity as for BG505. Meanwhile, T estimates of JR-FL were at least seven if the reported mean is accurate and showed elevated trends with increasing variation (Fig. 6C). Again, S and T exhibited a strong positive correlation of 0.7701 (Fig. 6B and C). For L193A JR-FL, the estimate of T = 9 (Fig. 6D) is higher than T of 7 for wild type, while the S is equivalent. S of 2 appeared in those areas where T estimates are relatively large, dictated by a large mean with a low-deviated number of trimer distributions (Fig. 6E and F). Regardless of wild-type or more open L193A JR-FL, S and T were positively correlated (Fig. 6). Under most cases of virion trimer number distributions, T estimates of L193A JR-FL were relatively larger than those of wild type, whereas the estimates of S were similar. Our findings hint that Env exhibiting a propensity to sample fusion downstream conformations may need more trimers for HIV-1 entry than that of predominantly closed Env.

## DISCUSSIONS

### HIV-1 entry stoichiometry and challenges of its determination

HIV-1 entry stoichiometry defines the number (T) of required interacting Env molecules with cell receptors for virus entry to infect the host and sets the threshold to differentiate noninfectious virions (less than T functional trimers) and infectious virions in the viral pool (20–23). To prevent or restrict virus entry, entry stoichiometry informs the minimum number of trimers on a virion that needs to be blocked by neutralizing antibodies, small molecules, or fusion inhibitors (18, 20) to set bounds for the number of functional ones below T. The stoichiometric requirements of HIV-1 entry are not completely resolved, as they are for most other biological processes involving membrane fusion, in part due to technical and experimental limitations. Direct counting Env trimers in an approximate way has been attempted in a limited number of studies using electron microscopes (16, 27–29, 34, 35). Visualization of 5–7 rod-like shape density at the virus–cell contact zone (35), termed the “entry claw,” was a point of reference for researchers to describe the spatial context of potential stoichiometry. However, technical limitations have prevented us from being definite about whether the rod-like shape represents the Env trimer observed by 3D electron tomography (35), and so do the 2–3 “spokes” seen by 2D electron microscopy as putative fusion intermediates (34). Despite the uncertainties, the "entry claw" formed by 5–7 Env trimers implied the stoichiometry of at least 5–7 trimers for the tested strain due to the use of fusion inhibitors and the uncertainty of whether these trimers were functional. Recently, a cryo-ET study of Env–CD4 interactions captured massive Env–CD4 clusters at the membrane–membrane interface (16), suggesting a great number of Env trimers engaging with CD4 molecules in the fusion activation process. While direct counting trimers at the fusion interface remains technically challenging, combining heterotrimer virion infectivity with mathematical modeling has provided a practical way to estimate entry stoichiometry (20, 21). The distinct T values were subsequently given in various studies (19, 21–25). The discrepancy is likely attributed to differences in both models (such as stoichiometric parameters and assumptions on trimer numbers/distributions per virion) and experiments (the use of different entry-deficient mutants and deviations in infectivity measurements), especially under the lack of information on trimer number distributions per virion.

### Estimation of entry stoichiometry, where we are now

**Fig 7:**
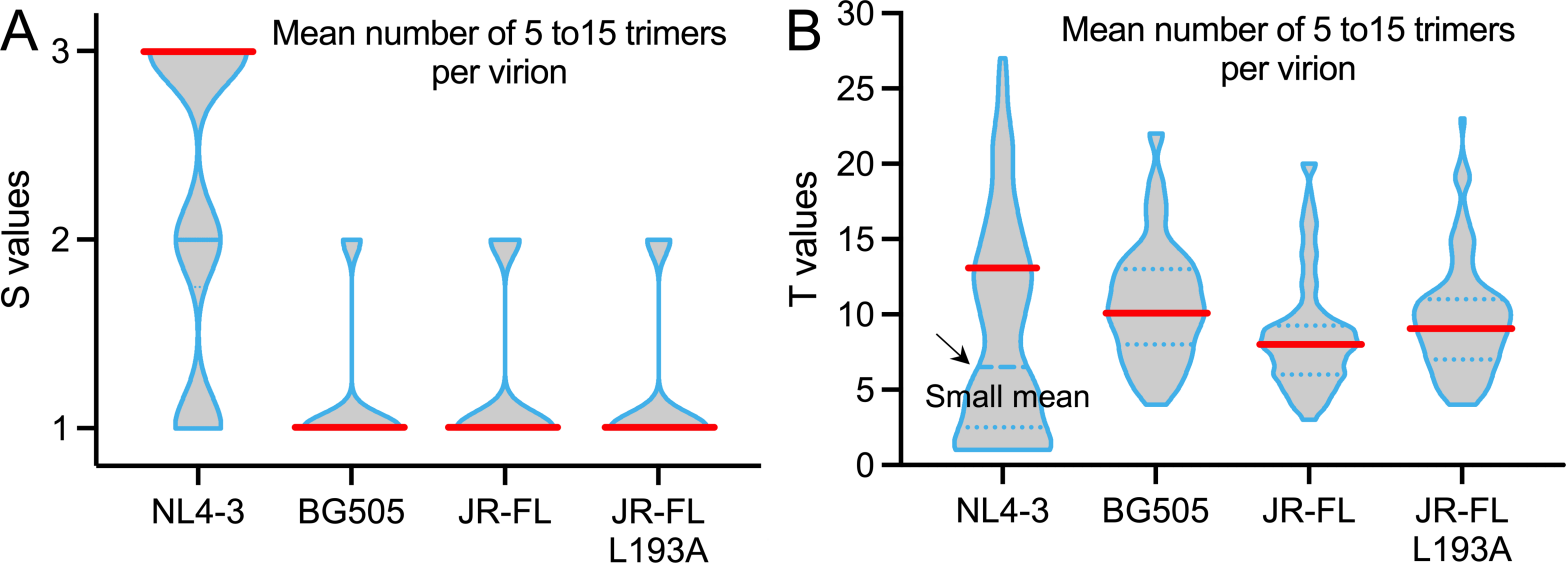
Distributions of estimated S and T across the generally believed range of average virion trimer numbers of 5 to 15. (**A**) Violin plot of inter-protomer opening cooperativity S estimates for tested Env strains. Red lines indicate the prevalent S. (**B**) Violin plot of entry stoichiometry T estimates for tested Env strains. Red lines indicate the reasonable on-average T values in dominance. Clusters of small T values of NL4-3, as indicated by a black arrow, come from the scenarios of much smaller mean numbers than the reported 14.

Despite the unreconciled estimates of T, continuous efforts have been made to achieve accuracy in T approximation from mathematical and experimental aspects (19, 21–25). It was exemplified in a most recent study by Brandenberg et al., in which they comprehensively evaluated T of 11 Env strains based upon a basic model and their experimentally determined trimer numbers for all tested strains (19). The intrinsic variation in T across multiple Env strains was first observed (19), which agrees with our estimate of substantially different T values across three tested strains and one variant (Fig. 4 to 6). Similarly, we also showed that trimer number distributions per virion play a pivotal role in estimating T for Env strains (Fig. 1 to 6). For a parallel comparison, we initiated our extended model with the pre-determined reported distributions of trimer numbers, but our model yielded larger T values of BG505, NL4-3, and JR-FL (Fig. 4A, 5A and 6A). The differences could be due to the consideration of opening cooperativity, the use of replication-competent virions, and the D368R mutant in our study, as mentioned earlier. Our estimate of T for NL4-3 is no less than 13 if there is an average number of 14 NL4-3 trimers per virion (Fig. 7). The number of Env trimers on HIV-1 virions is generally believed to range from 5 to 15 (27–29). If so, our estimates for BG505 and JR-FL are, on average, no less than seven (Fig. 7). While NL4-3 needs more trimers for entry than BG505 and JR-FL, which is consistent with the prior study, these actual numbers from our analysis are still much larger than previously suggested (19). The number of more than seven for primary isolates BG505 and JR-FL seems in line with the potential 5–7 trimer “entry claw” (35). As discussed earlier, statistically, T has to be larger than the number of clustered trimers captured by the fusion inhibitor. Our estimates of larger T values for tested Env strains are also consistent with recent cryo-ET observations of Env–CD4 clustering and ring formation at the interface between two membranes (16). Of note, without knowing functional trimer number distributions per virion, it remains challenging to give a precise estimation of T for any strain.

To advance, we extended our model from a single estimation to, for the first time, generate distributions of T values and display them on a map of virion trimer number distributions. The predicted T maps (Fig. 4D, 5D, 6C and D) by our model offer a unique angle to peer into potential changing directions or trends of T as distributions of virion trimer numbers differ. The estimates of T range from 1 to 30, varying profoundly with virion trimer distributions (Fig. 4 to 6). The wide range of T argues that the discrepancy in T values estimated in previous studies may partly be caused by the assumed or measured trimer number or distributions in models. Besides mapping S and T on trimer number distributions, the merits of our study also involve using replication-competent virions, choosing closely relevant CD4-binding deficient D368R to the definition of T, factoring floating trimer number distributions and opening cooperativity in modeling. Another important merit is unraveling the positive correlation between opening cooperativity and entry stoichiometry, as discussed in the next paragraph. The limitation of our model is that it can not exclude the possibility that other biologically relevant stoichiometric parameters or experimental conditions not considered in this study can potentially shape the values of paired S/T even further. Such factors, for example, are inter-Env distances, opening cooperativity across different trimers, gradual effects of cooperativities, processed vs unprocessed Env, different T/F Env strains, the use of primary CD4+ T cells or macrophages for infectivity, more open Env mutants, etc. While we acknowledge that our descriptive model is simplified and has limitations, we would like to clarify that it is designed to understand, summarize, and characterize our experimental data, offering insights into underlying patterns and relationships rather than imposing them. We would also like to clarify that a higher or lower absolute value of T does not directly imply correlation strength with S, as the correlation coefficient reflects the spread and the trend of data points across groups. Our findings consistently demonstrate a positive correlation between T and S, which we report objectively without overinterpreting the coefficient value. Thus, we anticipate that the correlation between S and T will be preserved. The paired S and T outputs of our mathematical model are directly determined by the model inputs, specifically the infectivity data points that define the curve (i.e., the curve shape). The consistent positive S–T correlation observed across a broad range of model inputs, including diverse infectivity curves with four distinct shapes from three different Env strains and one open variant, strongly validating the key finding of this study: a positive correlation between S and T. Thus, while the shapes of infectivity curves may vary under different experimental conditions, the positive correlation is expected to persist.

### A closer look at Env cooperativities

Env trimers on virions are cooperative in multiple ways. Entry stoichiometry (T) depicts multiple trimers working concertedly to execute a biology process, representing inter-trimer cooperativity. Moving our focus to the individual protomers within any trimer, how three protomers work together (S, the inter-protomer opening cooperativity) is another means of Env coordination. It was described in some earlier studies as cooperative subunits or subunit stoichiometry (22, 36–38). Inter-protomer opening cooperativity was explicitly indicated in the asymmetric opening of the trimer triggered by 1, 2, or 3 CD4 molecules (10, 16, 17). The opening of Env trimers does not necessarily need three CD4 molecules, as a CD4-bound protomer (s) was suggested to be able to release the conformational constraining domain elements of the neighboring one to loosen the trimer (10, 16, 17). Different degrees of such opening cooperativity or compensation are also evident in the observation that M-tropic HIV-1 can infect cells using just one CD4 molecule (39), while T-tropic needs to have multiple CD4 (39) along with multiple CCR5 molecules (40). The compensatory effects within three protomers in the estimation of Env trimers T were explored in a previous mathematic modeling study (24). In this work, they performed model analyses of published infectivity data from earlier reports (21, 25), in which the entry-deficient mutants V513E and R508S/R511S were used. These mutants are not directly associated with CD4 binding and, therefore, may not readily inform opening compensation, as observed with D368R in our study. Using a liminal model (where a virion is considered infectious if it contains at least T functional trimers, like our model), the study estimated T to be four or 5, based on the assumption that each virion harbors a fixed total of nine trimers. In contrast, instead of assuming a constant number of trimers per virion, our model accounts for virion trimer number distributions and predicts 2D S–T distributions across a map representing a broad range of virion trimer number distributions (Fig. 3 to 6), adding depth and flexibility to the analysis.

In our study, we integrated inter-protomer opening cooperativity into our estimations of entry stoichiometry that reflects inter-Env cooperativity (Fig. 2). Inter-protomer opening cooperativity S in our model is limited to intra-trimer, which does not include opening cooperativity across different trimers that is more complicated due to trimer–trimer distances and membrane fluidity. Model fitting of our experimental results further revealed the strikingly positive correlation between S and T for all tested Env strains or constructs (Fig. 4C, D, 5C, D and 6). Tier 1 Env NL4-3 is neutralization-sensitive, whereas Tier 2 Env strains BG505 and JR-FL are naturalization-resistant (41, 42). The observed high opening compensation of NL4-3 (Fig. 7) seems to align with Env trimers exhibiting more or easily open conformations of neutralization-sensitive Tier 1 Env strains (41). NL4-3 trimers are generally believed to have a high propensity for spontaneous opening, a characteristic that is relevant to the discussion of defective protomers and cooperativity. This spontaneous opening tendency does not contradict the cooperative CD4-induced inter-protomer opening central to our analysis. BG505 and JR-FL Env exhibited low and intermediate opening compensation within realistic virion trimer number distributions of mean ranging from 5 to 15 (Fig. 7). This phenotype is likely related to the propensity of Tier 2 Env trimers to be in a closed conformation (41), which may require more energy to compensate for opening the CD4-binding defective protomer by the neighboring functional protomer. In accord with this energy aspect, a more open JR-FL L193A appeared to need more Env trimers for entry than that of wild type (Fig. 7). Our results imply that Env trimers with high inter-protomer opening cooperativity tend to require more trimers for viral entry. This striking correlation supports the thermodynamics aspect of viral membrane fusion. If three protomers are highly cooperative in the trimer opening, the energy needed to open up Env proceeding to post-fusion conformation may be less than that with weak opening cooperativity. Assuming the total energy required for viral membrane fusion is constant or similar across different strains, high S will be combined with more Env trimers for a productive fusion event. Our results suggest the likelihood of an intrinsic balance of Env cooperativity during viral entry.

In summary, we considered inter-protomer opening cooperativity and virion trimer number distribution in estimating entry stoichiometry after weighing biological reality and the complicity of modeling. We included heterotrimer replication-competent viruses for infectivity model inputs. Our analysis revealed that stoichiometry greatly depended on virion trimer number distributions, and values varied for different strains. Our results highlighted a remarkable positive correlation between the degree of inter-protomer opening cooperativity and the required number of functional Env trimers for entry. The positive correlation persisted in all tested Env strains. This finding suggests an inherent equilibrium of Env cooperative interactions; that is, virions with easily open Env (high S) may need to recruit more Env trimers (large T) to compensate for the total energy for fusion. The stoichiometry of HIV entry is one of the key regulators in virus infectivity, and its estimation contributes to a better understanding of virus transmissibility and susceptibility to inhibition by neutralizing antibodies or entry inhibitors. Our findings thus may aid future approaches in HIV vaccine or entry inhibitor design.

## MATERIALS AND METHODS

### Cell lines

HEK293T cells (human embryonic kidney) were obtained from ATCC (CRL-3216), and TZM-bl cells were obtained from the NIH AIDS Reagent Program (ARP-8129). HEK293T cells were used to produce replication-competent and pseudotyped HIV-1 viral particles, and TZM-bl cells were used as target cells for quantifying viral infectivity. These two cell lines were cultured in high-glucose Dulbecco’s Modified Eagle Medium (DMEM, Gibco, Cat #11-965-092, Waltham, MA, USA) containing 10% (v/v) heat-inactivated fetal bovine serum (FBS), 100 U/mL penicillin-streptomycin (PS), and 2  mM L-glutamine. Cells were maintained in a 37°C tissue culture incubator with 5% CO_2_.

### Plasmids

This study used plasmids that separately encode three Env strains, including BG505, JR-FL, and NL4-3. BG505 and JR-FL are clinical neutralization-resistant Tier 2/3 strains, in which BG505 is a clinical Clade A transmitted/founder strain, and JR-FL is a Clade B strain. NL4-3 is a laboratory-adapted neutralization-sensitive Tier 1 strain. Plasmids encoding HIV-1_Q23_ Env_BG505_ (43, 44) and pNL4-3 virions were used to make replication-competent virions. pNL4-3 deltaEnv encoding viral backbone and pCAGGS JR-FL encoding Env_JR-FL_ or Env_JR-FL L193A_ were paired to make pseudotyped virions. The CD4-binding defective D368R Env plasmids were constructed by introducing D368R mutation into replication-competent pNL4-3, HIV-1_Q23_ Env_BG505_, and Env-expressing pCAGGS JR-FL or JR-FL L193A by site-directed mutagenesis according to the manufacturer instructions. The presence of the desired mutations and the absence of unintended coding changes were confirmed by Sanger sequencing or next-generation sequencing. An intron-regulated luciferase plasmid (HIV-1-InGluc) that encodes Gaussian luciferase was used as a reporter.

### Viral infectivity assay

The preparation of replication-competent or pseudotyped virions and quantification of titers on TZM-bl cells were as previously described (45–47). Replication-competent NL4-3 virions surface-incorporated with mixed trimers containing wild-type and D368R Env were generated by co-transfection of HEK293 cells with wildtype NL4-3 and mutant Env at serial ratios and HIV-1-InGluc using polyethyleneimine or Fugene 6. In this paper, mixed trimers are interchangeable with heterotrimers. The ratios of wild-type and mutant Env used were 10:0, 9:1, 8:2, 7:3, 6:4, 5:5, 4:6, 3:7, 2:8, 1:9, and 0:10. Replication-competent HIV-1_Q23_ Env_BG505_ were produced similarly. Pseudotyped virions carrying mixed JR-FL (or JR-FL L193A) trimers were generated by co-transfection of HEK293 cells with a mix of pCAGGS JR-FL wild-type and mutant Env at serial ratios, along with backbone NL4-3 deltaEnv and reporter HIV-1-InGluc. At 40 h post-transfection, virions carrying mixed Env trimers were harvested, filtered through 0.45 µm filters, and added to TZM-bl cells stably expressing CD4 and CCR5/CXCR4. At 48 h post-infection, viral infectivity was further quantified by measuring Gaussian luciferase activity. Relative viral infectivity was calculated by normalizing relative light units to virions produced solely with wild-type Env-encoding plasmids. Relative viral infectivity was displayed as a function of the fraction of CD4-binding defective D368R mutant Env-encoding plasmids. For each tested Env strain, the relative infectivity curve was further fitted by mathematic models to estimate entry stoichiometry (T) and the degree of inter-protomer opening cooperativity (S).

### Trimer number distributions on virions

Trimer number distributions per virion are assumed to follow discretized Beta distribution, in which two shape parameters were converted to be expressions of average or mean trimer numbers (mean, μ) and standard deviation (std, δ) or variance (var, δ^2^). Both μ and δ parameters are integers, representing average and deviated counts of Env trimers on a virion. Therefore, discretized Beta distributions can vary with pairs of mean and deviation of your choice. In estimating a single number of T and S, the mean trimer number μ used the experimentally determined counts for each tested Env strain in a previous report (19), and δ was set as a square root of 49/14*μ for consistent comparison. However, our model is not limited to any single count of mean or deviation. Instead, the heatmap of S or T distributions displayed matrices of S or T estimates as a function of discretized Beta (μ, δ) with no constraints between two parameters, in which μ ranges from 1 to 30 and δ from 1 to 10 (var: 1 to 100). Therefore, the heatmap can describe the exploration of S or T with all possibly realistic combinations of Beta (μ, δ).

### Modeling of the interplay between trimer number distribution, infectivity, and T

To model the interplay, we constrained our Beta distribution with one parameter mean, and the other parameter standard deviation (std) was set as a square root of 49/14*μ. The purpose of this model is to exemplify the relationships between trimer number distribution, viral infectivity, and the entry stoichiometry T. How std was chosen would not matter to their intercorrelation (positive or negative). Theoretically, given a pre-defined trimer number distribution, the infectious virions are those having equal or more than T trimers on the virion. Therefore, those qualified virions can contribute to infectivity, and their total count can be displayed as a percentage of the entire virions pool. As trimer number distributions per virion vary, the degree of T affecting infectious virions in the population will change. In a similar way, infectious virions in the population can be displayed over trimer number distributions, and they vary with T numbers.

### Overall description of simultaneous estimates of T and S

The mathematical models we have developed are crucial in understanding and predicting the relative infectivity (RI) of mixed-Env replication-competent and pseudotyped virions. We aim to use these models to make considerable predictions regarding the RI of mixed-Env virions after considering the combinatorial aspects of the entry process. The mathematical modeling was inspired by a basic model (19, 23) and expanded by relaxing trimer number distributions as well as introducing S. Combinatorial aspects of our model primarily involve the distributions of Env trimer numbers per virion, entry stoichiometry T, and inter-protomer opening cooperativity (or compensation) S that were often overlooked. Our modeling process is based on experimental and mathematical assumptions (see following Methods) to explore the best fit of experimental RI data under no constraints or pre-experimentally constrained trimer number distributions. Experimental assumptions in generating RI of mixed-Env replication-competent or pseudotyped virions include (i) each functional Env trimer on the viral surface contributes equally to the infectious virions in the population, (ii) the cell-transfection efficiency of wild-type Env and mutant Env plasmids are the same, (iii) there is no obvious difference in virion Env incorporation level of wild-type and D368R Env, which we previously confirmed for NL4-3, JR-FL, and BG505 Env by Western blotting (10), (iv) wildtype and D368R Env proteins are assembled randomly and independently into trimers with equal possibilities.

### Mathematical models of RI of heterotrimer virions

Equation (1) describes the overall mathematical model, which is derived from equations (2)-(6)-6.


(1) RI(fm,S,η,T)=∑N=TNmax{ηN∑g=TN(Ng)pg(1−p)N−g}∑N=TNmaxηN

Parameters in equation (1) include:

***RI*** is the relative infectivity, ***f_m_*** is the fraction of mutant Env D368R,

***S*** represents the discrete degrees of inter-protomer functional compensation, reflecting inter-protomer opening cooperativities, as an integer of 1, 2, or 3,

***T*** is the entry stoichiometry (the number of Env required to interact with CD4 for HIV entry),

$\eta_{N}$ is the probability of a virion having ***N*** trimers (the max ***N^max^*** is set to 100, and the number of trimers follows a Beta distribution), and

***g*** is the number of functional trimers within ***N*** trimers,

Equation (1) is derived from consecutive steps involving the following (i) trimer number distribution, (ii) probability of functional trimers, and (iii) ***RI*** as a function of ***T***, ***S***, trimer distribution **Beta** (μ, δ), and ***f_m_***.

#### Trimer number distribution on virions

We assume that the number of functional Env trimers on virions follows discretized Beta distributions, in which two shape parameters are expressed with the average number (mean μ) of functional trimers and the deviation (δ) or variance (δ^2^). Therefore, the Beta distribution can be treated as a function of **Beta** (μ, δ). The maximum number of functional Env trimers per virion (***N****_max_*) is set to be 100.

#### Probability of an Env trimer of protomers being functional

***p*** is the probability of a trimer being functional, contributing to infectivity. The probability of having D368R in an Env trimer follows a binomial distribution with a trial number three and a success parameter ***f_m_***. In experiments, ***f_m_*** is the fraction of mutant D368R Env. We use three scenarios (***S*** = 1, 2, and 3) to represent the discrete degrees of functional compensation, translating to inter-protomer opening cooperativity/compensation in our case.

In the case of ***S*** = 1, a single D368R mutant protomer renders the whole trimer unfunctional. The probability (*P*) of a trimer being functional can be described mathematically as the probability of three successes in three Bernoulli trials with a success rate of 1 ***−f_m_***.


(2) $$p={\left(1-fm\right)}^{3}=1-3fm+3fm^{2}-fm^{3}$$

In the case of ***S*** = 2, two D368R mutant protomers render the whole trimer unfunctional. Similarly, the *P* can be expressed as:


(3) $$p=1+2fm^{3}-3fm^{2}$$

In the case of ***S*** = 3, three D368R mutant protomers render the whole trimer unfunctional. Then, the *p* can be expressed as equation (4):


(4) $$p=1-fm^{3}$$

#### RI as a function of T and S

If each virion has a constant number (***N***) of Env trimers from a Beta (μ, δ), the probability of each virion carrying an exact number of ***g*** functional trimers is:


(5) (Ng)pg(1−P)N−g

The expression in equation (5) is the basic binomial probability of ***g*** successes in ***N*** trials with success probability ***p***.

Given the entry stoichiometry ***T***, the ***RI*** of virions having *N* trimers is the cumulative probability that a virion has more than or equal to ***T*** trimers:


(6) RI=∑g=TN(Ng)pg(1−p)N−g

When ***N*** varies, ηNis the probability of a virion carrying ***N*** trimers, ***N*** ranges from 0 to *N^max^*. ***RI*** can be further expressed in equation (1) as a function of S, T, and Env trimer number distributions.

### Estimation of T and S matrices as a function of virion trimer number distributions

Without assuming an exact trimer number distribution Beta (μ, δ), we predicted RI curves of mixed trimer virions under varying Beta (μ, δ). We then explored the best fits of experimentally observed RI data by evaluating the residual sum of squares (RSS) between experiment-observed and model-predicted RI data. For each experimental RI curve, our model simultaneously generated a T matrix and an S matrix, corresponding to any given combination of integer μ and δ of a Beta distribution. It is generally believed that each virion may have 5 to 15 Env trimers. Thus, we set a wide range of the mean μ from 1 to 30 with a standard deviation of 1 to 10. Given any preset Beta (μ, δ), our model will find the best fits of S and T. The best fits were further validated by Bootstrap sampling with 10,000 replicates. The heatmaps of S and T were made using Prism 10 to display the matrix data of S and T estimated in MATLAB. All model analyses were performed using customized MATLAB scripts, available upon request.

### Statistical analyses

Statistical correlation analyses of S and T were done using the Pearson built-in function (a measure of their linear dependence) in MATLAB. Quantitative graphs of experimental data were analyzed using Prism 10 (GraphPad).

## SUPPLEMENTAL MATERIAL

Supplemental Figures mbio.02754-24-s0001.pdf Figures S1 and S2.

ASM does not own the copyrights to Supplemental Material that may be linked to, or accessed through, an article. The authors have granted ASM a non-exclusive, world-wide license to publish the Supplemental Material files. Please contact the corresponding author directly for reuse.
