# Supplementary material for: Inter-protomer opening cooperativity of envelope trimers positively correlates with HIV-1 entry stoichiometry
Source: mBio. 2025 Feb 25;16(4):e02754-24. doi: 10.1128/mbio.02754-24 (PMC11980385; doi:10.1128/mbio.02754-24)
Supplement: Supplemental Figures — Figures S1 and S2. [file mbio.02754-24-s0001.pdf]

Supplementary Material for

Inter-protomer Opening Cooperativity of Envelope Trimers Positively Correlates with HIV-1 Entry Stoichiometry

Running title: Linking Inter-protomer to Inter-Env Cooperativities

Revansiddha H. Katte[1], Wang Xu[1], Yang Han[1], Xinyu Hong[1]*, and Maolin Lu[1]*

[1]Department of Cellular and Molecular Biology, School of Medicine, The University of Texas at Tyler Health Science Center, Tyler, Texas, USA

*Correspondence to: Xinyu Hong (xinyu.hong@aya.yale.edu) and Maolin Lu (maolin.lu@uthct.edu).

Includes Supplementary Figures

Figure S1
Figure S2

Figure S1.

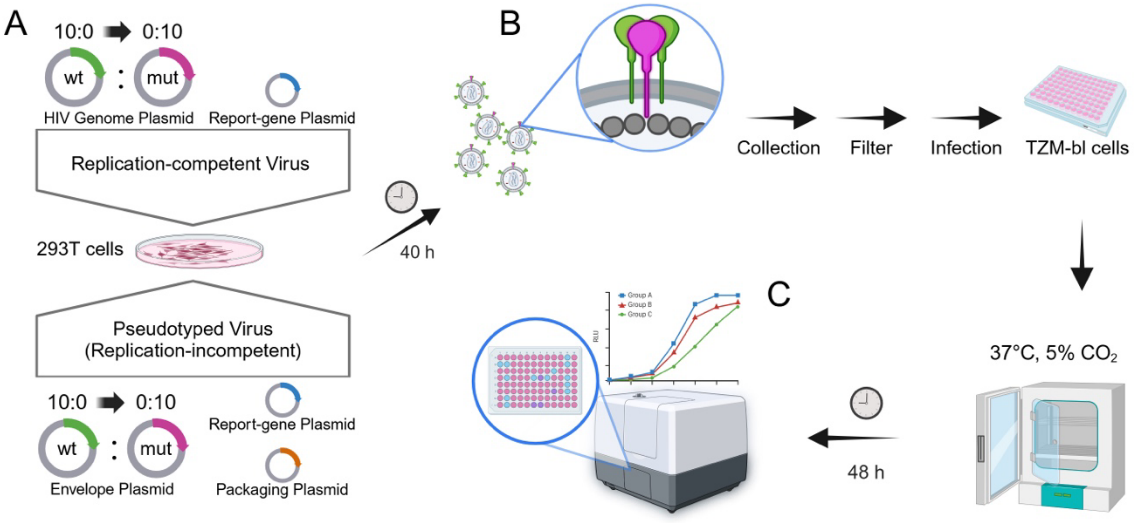

Fig S1. Schematic representation of the experimental design.
(**A**) HEK293T cells were used for co-transfection and to produce replication-competent (up) and pseudotyped (down) HIV-1 viral particles.
(**B)** Viruses carrying mixed Env trimers (or heterotrimers) were harvested 40 hours after transfection, filtered, and titered on TZM-bl cells.
(**C)** A Gaussia luciferase assay was used to quantify virus infectivity 48 hours after infection.

Figure S2.

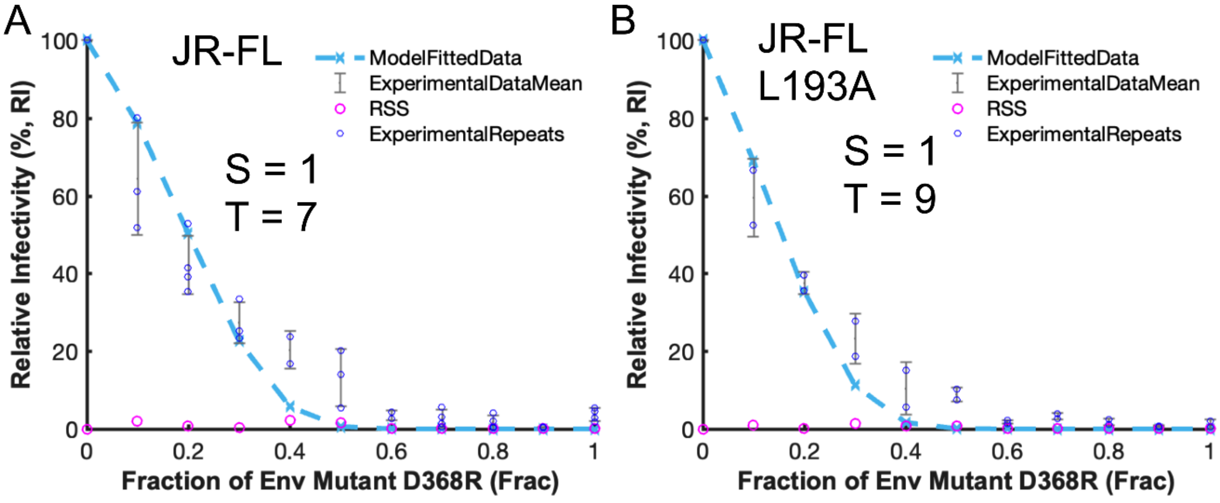

Fig S2. Curve fitting of RI of pseudotyped virions carrying $Env_{JR\text{-}FL}$ wildtype or L193A.

(**A**) Estimates of S=1 and T=7 for $Env_{JR\text{-}FL}$ from model fits of relative virus infection of pseudo typed virions carrying $Env_{JR\text{-}FL}$ over the fraction of D368R $Env_{JR\text{-}FL}$ mutant, given reported trimer distribution Beta (12, sqrt (42)) of $Env_{JR\text{-}FL}$.

(**B**) Estimates of S=1 and T=9 for $Env_{JR\text{-}FL}$ L193A.
